# Comment on Reiss et al. The Effect of Androgen Deprivation Therapy on the Cardiovascular System in Advanced Prostate Cancer. *Medicina* 2024, *60*, 1727

**DOI:** 10.3390/medicina61101723

**Published:** 2025-09-23

**Authors:** Mike Ufer, Viatcheslav G. Rakov

**Affiliations:** Sumitomo Pharma Switzerland GmbH, Aeschengraben 27, CH-4051 Basel, Switzerland

In a recent review article, Reiss A.B. et al. thoroughly addressed the effect of androgen deprivation therapy (ADT) on the cardiovascular system in advanced prostate cancer [1]. The authors comment on lower cardiovascular event rates of the gonadotropin-releasing hormone (GnRH) antagonists relugolix and degarelix in comparison to GnRH agonists. In addition, they mention relugolix as the only oral GnRH receptor antagonist approved for treatment of advanced prostate cancer, considering some of its properties as favorable compared to injectable GnRH agonists or antagonists such as degarelix (e.g., absence of injection site reactions and more rapid onset and recovery of testosterone suppression) [2,3,4].

In terms of effects on cardiac repolarization, the authors state that “in contrast to relugolix and leuprolide, degarelix is not associated with QT prolongation” [1]. This statement is based on the results from a randomized, placebo- and active comparator-controlled single-dose TQT study with degarelix in 80 healthy men [5]. In this cross-over study, degarelix did not exhibit relevant QT prolongation at supratherapeutic exposure following the administration of a single, intravenous dose [5]. Despite this negative single-dose TQT study with degarelix, it should be noted that QT prolongation is a class-related risk of ADTs due to testosterone suppression and is applicable to chronic treatment with any GnRH agonist or antagonist, including degarelix [6]. Mechanistically, testosterone suppression is associated with a prolonged action potential duration in the heart muscle due to reduced expression of potassium channels and an upregulation of L-type calcium channels [7]. Therefore, we believe that the results of this single-dose TQT study do not justify the authors’ statement claiming the absence of any QT prolongation risk for degarelix, as this depends on exposure duration and the extent of testosterone suppression. In line with our view, the authors themselves state elsewhere in their article that the effect of QT prolongation “is seen with both GnRH agonists and antagonists and is thought to be a result of medical castration” [1].

In the context of relugolix, a dedicated TQT study was also conducted as part of its clinical development program. In this randomized, double-blind, parallel-group study, 280 healthy male and female subjects received a single therapeutic or supratherapeutic dose of relugolix, moxifloxacin (=positive control), or a placebo. The upper bound of the 95% CI for ΔΔQTc was less than 10 ms at all timepoints, indicating the absence of relevant QT prolongation [8,9] in line with the results of the TQT study with degarelix [5]. The results of this negative TQT study with relugolix are included in its labeling information, but it is also stated that ADTs such as relugolix may prolong the QT interval, and health care providers should consider whether the benefits of ADT outweigh the risks in patients susceptible to QT prolongation [8,9].

In summary, we believe that the article by Reiss AB et al. is misleading by indicating that no QT prolongation potential applies to degarelix, whereas this would apply to relugolix despite consistent negative results from the single-dose TQT studies with degarelix and relugolix. Although there is, to our knowledge, no positive evidence for such risk from placebo-controlled, long-term safety studies, the labeling information of both relugolix and degarelix includes a similar warning of QT prolongation, as it is a well-established risk associated with ADT secondary to testosterone suppression [8,9,10,11].
